# Reciprocal associations between job strain and depression: A 2‐year follow‐up study from the Survey of Health, Ageing and Retirement in Europe

**DOI:** 10.1002/brb3.1381

**Published:** 2019-08-25

**Authors:** Ya‐Mei Qiao, Ya‐Ke Lu, Zhen Yan, Wu Yao, Jin‐Jing Pei, Hui‐Xin Wang

**Affiliations:** ^1^ Department of Occupational and Environmental Health Sciences College of Public health Zhengzhou University Zhengzhou China; ^2^ Department of Occupational and Environmental Health Sciences School of Public Health Hainan Medical University Haikou China; ^3^ Stress Research Institute Stockholm University Stockholm Sweden; ^4^ Aging Research Center Department of Neurobiology, Care Sciences and Society Karolinska Institutet Stockholm Sweden

**Keywords:** depression, job strain, older workers

## Abstract

**Background:**

A growing number of people suffered from depression. This study examined the depression prevalence in workers across 10 European countries plus Israel and the reciprocal associations between job strain and depression.

**Methods:**

The study population consisted of 7,879 workers aged 50–63 years at baseline (2004) from the Survey of Health, Ageing, and Retirement in Europe (SHARE). Job demands (physical or psychosocial) and job control variables were derived from the Job Content Questionnaire (JCQ). Two 4‐category job strains (physical and psychosocial) were obtained based on the cross‐tabulation of these dichotomized demands and control variables. There were 4,284 depression‐free, 3,259 high physical strain‐free and 3,195 high psychosocial strain‐free participants at baseline who were followed up for 2 years to detect incident depression, high physical job strain, or high psychosocial strain, respectively. The reciprocal associations between job strain and depression were analyzed by multivariate logistic regression and multivariate multilevel logistic regression adjusting for potential confounders.

**Results:**

The prevalence of depression varied from the lowest 12.5% in Germany to the highest 27.2% in France. Compared to individuals with low strain, a significantly higher risk of depression were found in individuals with high physical strain (OR = 1.39) and high psychosocial strain (OR = 1.55), after adjusting for potential confounders. Depression at baseline was not significantly associated with subsequent high job strain. Similar results were observed from multilevel models that took into consideration of the potential country‐level influences.

**Conclusions:**

The prevalence of depression varies across countries in Europe. Avoiding high job strain may be an effective preventive strategy to prevent depression epidemic.

## 1 INTRODUCTION

Depression is a common and serious emotional disorder that causes feelings of sadness and/or a loss of interest in activities for weeks, months, or even years. It can lead to a variety of emotional and physical problems and decrease a person's ability to function at work and at home. According to the statistics of World Health Organization (WHO), 350 million people are expected to suffer from depression worldwide in 2030 and it will emerge as a major contributor to the global burden of disease (WHO, 2017). A wide range of depression prevalence has been reported across age groups from 1.8% of children aged 8–11 years to 27% of population aged over 60 years (Castro‐Costa et al., 2008; Polainolorente & Domènech, 1993). The prevalence figures also differed in countries, from 5%–7% in Japan to 31% in the USA (Blackmore et al., 2007; Heim, Wegmann, & Maercker, 2017), with European countries in the middle, for example, Hungary (7.1%), Austria (7.6%), Romania (7.6%), Estonia (7.9%), Ireland (8.5%), Spain (8.6%), Italy (9.2%), Slovenia (11.4%), Germany (12.9%), and France (15.4%) (Aichberger et al., 2010; Balazs et al., 2012; Narayanan, Potthoff, & Guether, 2009). Due to the multifactorial influence and various diagnostic criteria used in different studies, it is difficult to clarify whether or not these differences are real, which highlights the importance of using uniformed information source to obtain comparable estimations.

The influence of work conditions on mental health has been extensively studied over the last decades. Specifically, an adverse effect of high job strain on depression has been widely accepted (Mezuk, Bohnert, Ratliff, & Zivin, 2011; Stansfeld, Shipley, Head, & Fuhrer, 2012; Van Den Bogaard & Henkens, 2018; Wahrendorf, Blane, Bartley, Dragano, & Siegrist, 2013), although a few other studies did not find such an association (Landsbergis, Schnall, Warren, Pickering, & Schwartz, 1994; Ylipaavalniemi et al., 2005). In addition, a study stressed that the relationships between work‐related factors and depressive disorder could not remain when individual tests and higher level (such as country) of measures were simultaneously taken into account in the analysis, suggesting the importance of employing multilevel analysis especially when the structure of data is hierarchical (Muntaner et al., 2006). To our knowledge, most previous studies on the topic have not taken into account the influence of higher level factors.

Furthermore, there were inconsistent evidences on whether depression predicts subsequent high job strain among older workers (De Lange, Taris, Kompier, Houtman, & Bongers, 2004; Ibrahim, Smith, & Muntaner, 2009).

To fill the gap in current literature, the aims of this study were (a) to estimate the prevalence and incidence of depression in 11 countries (10 European countries and Israel) using an uniformed assessment; (b) to explore the association between job strain and depression incidence taking into account country‐level influence; and (c) to investigate whether baseline depression predicts subsequent high job strain over a 2‐year follow‐up period.

## 2 METHODS

### 2.1 Study population

The study population was derived from the first and second waves of the Survey of Health, Ageing, and Retirement in Europe (SHARE; Börsch‐Supan et al., 2008, 2013; Börsch‐Supan & Jürges, 2005), a longitudinal survey that aimed to collect medical, social, and economic data on the population aged over 50 years from 10 European countries (Austria, Germany, Sweden, Netherlands, France, Spain, Denmark, Switzerland, Greece, Belgium) plus Israel. The survey was carried out longitudinally at 2‐year intervals based on a computer‐assisted personal interviewing technique (CAPI). The first wave (wave 1) was initiated during 2004–2005, and second wave (wave 2) took place in 2006–2007. The overall response rate was 48.2% and differed in participating countries: Austria (38.9%), Germany (49.9%), Sweden (45.4%), Netherlands (53.7%), France (70.1%), Spain (37.3%), Denmark (58.9%), Switzerland (32.8%), Greece (55.1%), Belgium (31.6%), and Israel (54.2%). Respondents who took part in both wave 1 (Börsch‐Supan, 2018a) and wave 2 (Börsch‐Supan, 2018b) of SHARE were chosen for our analyses.

In order to study the reciprocal associations between job strain and depression among working age population, the study consisted of participants aged 50–63 years at baseline, so that they would not reach retirement age of 65 after the 2‐year follow‐up (*n* = 13,823). Among them, 5,944 individuals were excluded due to missing information on job strain (*n* = 5,872) or depression (*n* = 72), leaving 7,879 participants to estimate the depression prevalence.

To study the association between baseline job strain and depression incidence developed during the 2‐year follow‐up, among 5,414 individuals both in wave 1 and wave 2, 77 individuals with missing data on depression at wave 2 and 1,053 individuals who had depression at baseline were excluded, leaving 4,284 depression‐free participants at baseline for the analysis.

To study the influence of depression on incidence of high job physically demands‐control strain, 1,086 individuals with missing data on job strain at wave 2 and 1,069 individuals who had high physical job strain at baseline were excluded, leaving 3,259 participants for the analysis.

To study the influence of depression on incidence of high psychosocial job demands‐control strain, 1,093 individuals with missing data on job strain at wave 2 and 1,126 individuals who had high psychosocial job strain at baseline were excluded, leaving 3,195 participants for the analysis.

**Figure 1:**
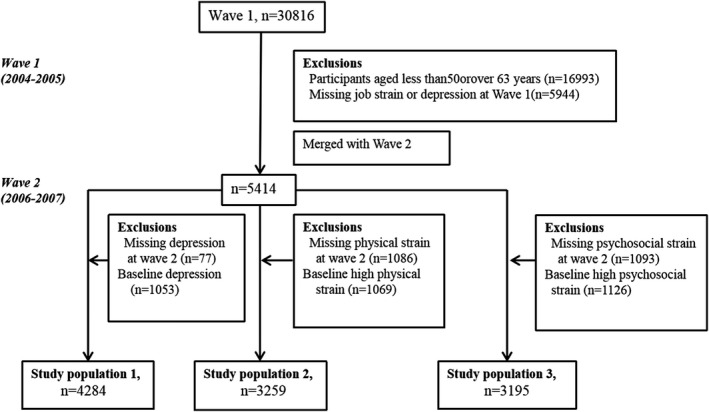
The study population. Study population 1: To study depression incidence. Study population 2: To study high physical job strain incidence. Study population 3: To study high psychosocial job strain incidence

Detailed flow diagram of the study participants was showed in Figure 1.

### 2.2 Assessment of depression

Depression was assessed by the Europe depression (EURO‐D) scale, a structured scale with 12 common depressive symptoms, and designed to be administered by trained lay interviewers. Participants were asked to answer the following questions with “yes” or “no”: depressed mood, pessimism, suicide, guilt, sleep, interest, irritability, appetite, fatigue, concentration, enjoyment, and fearfulness. The scores ranged from 0 to 12. Depression was defined as a score ≥4. Using this cut‐off, a reasonable sensitivity (63%–83%) and specificity (49%–95%) have been reported in studies carried out in 14 European countries (Prince et al., 1999), with a satisfactory internal consistency, ranging from 0.78 to 0. 95 (Castro‐Costa et al., 2008).

### 2.3 Assessment of job strain

Job Content Questionnaire (JCQ; Karasek et al., 1998) was one of the most widely used instruments to estimate job strain. In this study, job demands‐control model including four items derived from the JCQ was used (Van Den Berg, Schuring, Avendano, Mackenbach, & Burdorf, 2010). Physical demands were measured by a single item “My job is physically demanding,” and psychosocial demands were assessed by “I am under constant time pressure due to a heavy workload,” job control was assessed with two questions “freedom to decide how I do my work” and “the opportunity to develop new skills.” These items were quantified on a Likert scale with a score from 1 (strongly agree) to 4 (strongly disagree). The scores were summed up for each of the dimensions and dichotomized according to the country‐specific median value, which were similar to the overall median value of the participating countries. Individuals with scores above or below the median were regarded as high/low physical demands, psychosocial demands, and control, respectively.

Following the job demands‐control model, job strain was further categorized as a 4‐category physical strain and a 4‐category psychosocial strain based on the cross‐tabulation of their dichotomized job demands (physical or psychosocial demands) and job control variables: the 4‐category physical strain grouped as follows: (a) low physical strain (low physical demands + high control); (b) passive physical job (low physical demands + low control); (c) active physical job (high physical demands + high control); (d) high physical strain (high physical demands + low control); and a psychosocial strain: (a) low psychosocial strain (low psychosocial job demands + high control); (b) passive psychosocial job (low psychosocial job demands + low control); (c) active psychosocial job (high psychosocial job demands + high control); (d) high psychosocial strain (high psychosocial job demands + low control).

### 2.4 Assessment of sociodemographic and other potential confounders

Information on age, gender, education, body mass index (BMI), marital status as well as living arrangement, mobility limitation, chronic disease, self‐reported health, smoking, alcohol consumption, physical activity, and country were collected and taken into account as potential confounders in the study. Age and education were treated as continuous variables, and all other variables were treated as dichotomized variables in the analysis. Marital status and living arrangement were integrated into one variable, which was then dichotomized as married and living together with spouse/partner versus other categories (Van Den Berg et al., 2010). BMI was grouped into four categories: underweight (<18 kg/m^2^), normal (18–25 kg/m^2^), overweight (25–30 kg/m^2^), and obese (≥30 kg/m^2^). Country of residence was defined by their regular domicile in the respective SHARE countries.

Chronic disease was defined as the presence of any of the following diseases: heart attack, high blood pressure or hypertension, high blood cholesterol, a stroke or cerebral vascular disease, diabetes or high blood sugar, chronic lung disease, asthma, arthritis, osteoporosis, cancer, stomach or duodenal ulcer, peptic ulcer, Parkinson's disease, cataracts, hip fracture, and femoral fracture. Mobility limitation was defined as the presence of at least one of limitations in arm function or fine motor function, including walking 100 m, sitting for about 2 hr, getting up from a chair after sitting for long periods, climbing several flights of stairs without resting, climbing one flight of stairs without resting, stooping, kneeling, or crouching, reaching or extending your arms above shoulder level, pulling or pushing large objects like a living room chair, lifting or carrying weights over 10 pounds/5 kilos, like a heavy bag of groceries, and picking up a small coin from a table. Self‐reported health was grouped as good versus less than good.

Smoking was dichotomized as follows: smoker (currently smoking) and nonsmoker (never smoking daily for at least 1 year or stopped smoking currently), and alcohol consumption was dichotomized as follows: alcohol drinker (drinking >2 glasses of any alcohol beverages, like beer, cider, wine, spirits, or cocktails, almost 5–6 days a week or every day) and nondrinker (not drinking more than 2 glasses daily or 5–6 days a week). Physical activity was dichotomized as follows: vigorous or moderate physical activity versus less than moderate physical activity.

### 2.5 Statistical analysis

Chi‐square test was employed to examine baseline characteristics of the study population and depression incidence or high job strain developed during the 2‐year follow‐up, as well as the characteristics between participants and nonparticipants due to missing information. Univariate logistic regression was used to estimate crude odds ratio (OR) of the prevalence of depression in relation to individual characteristics and job strain status. Multivariate logistic regression was performed to estimate the OR of depression incidence in relation to baseline job strain, adjusting for age, gender, education, BMI, marital status, chronic disease, mobility limitation, self‐reported health, smoking, alcohol consumption, physical activity, and country. The same analysis was performed for high job strain incidence in relation to baseline depression.

Because individuals from the same country tend to be more alike in their working conditions and other characteristics than individuals from other countries, multilevel model was applied. This model allowed us to group individual influences within country, thus can include residual variations at both individual (level 1) and country levels (level 2). The following two models were fitted: (a) a random intercept‐only model (Model 0) to assess country‐level variation in depression incidence and (b) a model with both fixed and random intercepts and slopes (M1) to evaluate the impact of exposure, this model assumed the variables varying from country to country.

## 3 RESULTS

As compared to participants, nonparticipants were found to be more likely to be older than 60 years (18.16% vs. 11.53%) and to have higher levels of education (61.56% vs. 56.97%), less than good health (18.79% vs. 14.39%), and depression (38.87% vs. 13.96%; data not shown).

**Figure 2:**
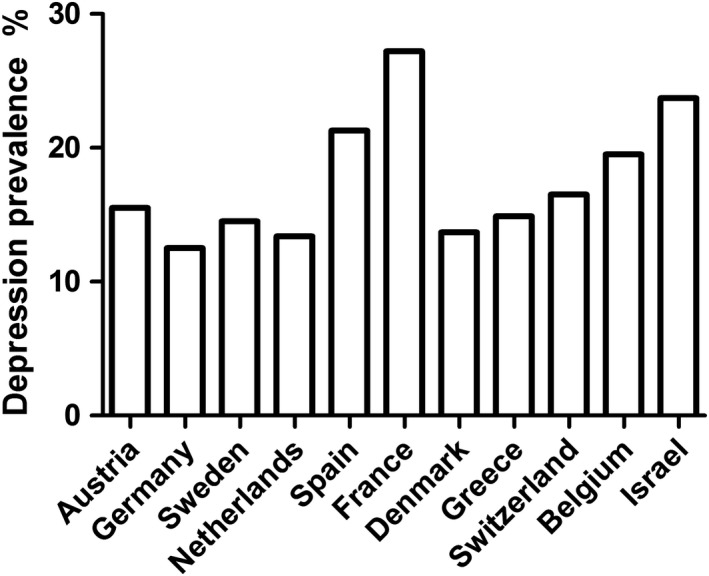
The depression prevalence in 11 countries

Table 1 shows the prevalence of depression and characteristics of the study population. The majority of the participants was aged 50–55 (47.72%), with similar number in both male and female. The prevalence of depression was 17.43% in this population, varying among countries and ranging from the lowest of 12.46% in Germany to the highest of 27.21% in France (Figure 2). We also found that participants were more likely to have depression if they were female (OR = 2.57), living alone (OR = 1.58), underweight (OR = 2.81), or obese (OR = 1.46), with any chronic disease (OR = 2.06), with at least one mobility limitation (OR = 3.07), with less than good health (OR = 3.57), with no physical activity (OR = 2.18), with high physical demands (OR = 1.14), high psychosocial demands (OR = 1.34), low job control (OR = 1.48), passive job (OR = 1.44), and high job strain (OR = 1.73). In contrary, older individuals (OR = 0.96), those with higher level of education (OR = 0.94), and alcohol consumers (OR = 0.82) were less likely have depression. When job strain was calculated based on the overall median of these countries, similar results were observed.

**Table 1:** Odds ratio (OR) with 95% confidence interval (95% CI) of depression in relation to baseline characteristics of the study population

Characteristics	Participants, *n*	Depression cases, *n*	Prevalence, 100 persons	OR (95% CI)
Age ($\bar{X}$ ± s)	55.2 ± 3.6	54.8 ± 3.5	–	0.96 (0.94–0.98)
Male	4,241	478	11.27	1.00
Female	3,638	895	24.60	2.57 (2.28–2.90)
Education ($\bar{X}$ ± s)	11.9 ± 3.9	11.1 ± 4.1	–	0.94 (0.92–0.95)
Marital status and living arrangement				
Married and living with a spouse	6,044	954	15.78	1.00
Living alone	1,835	419	22.83	1.58 (1.39–1.80)
BMI				
Normal	3,237	535	16.53	1.00
Underweight	126	38	30.16	2.81 (1.47–3.23)
Overweight	3,323	533	16.04	0.97 (0.85–1.10)
Obese	1,190	267	22.44	1.46 (1.24–1.72)
Chronic diseases				
No	3,283	385	11.73	1.00
Any diseases	4,596	988	21.50	2.06 (1.81–2.34)
Mobility limitation				
No	5,712	713	12.48	1.00
With ≥1 limitation	2,167	660	30.46	3.07 (2.72–3.46)
Health				
Good	6,224	857	13.77	1.00
Less than good	1,453	515	35.44	3.57 (3.13–4.06)
Smoking				
Nonsmokers	5,768	981	17.01	1.00
Current smokers	2,111	392	18.57	1.13 (0.98–1.27)
Alcohol consumption				
Nondrinker	6,884	1,223	17.77	1.00
Alcohol drinker	995	150	15.07	0.82 (0.68–0.99)
Physical activity				
Vigorous or moderate	7,622	1,295	17.99	1.00
Never	254	78	30.71	2.18 (1.66–2.86)
Physical demands				
Low	4,274	707	16.54	1.00
High	3,605	666	18.47	1.14 (1.02–1.29)
Psychosocial demands				
Low	3,950	605	15.32	1.00
High	3,929	768	19.55	1.34 (1.20–1.51)
Job control				
High	3,855	561	14.55	1.00
Low	4,024	812	20.18	1.48 (1.32–1.67)

Table 2 shows baseline characteristics of the depression‐free population and depression incidence developed during the 2‐year follow‐up. The incidence of depression was higher in female (13.09% vs. 7.02%), in those individuals with any chronic disease (10.78% vs. 8.05%) and poor self‐reported health (17.50% vs. 8.33%), and in those individuals who were physically inactive (16.83% vs. 9.45%) than their respective counterparts.

**Table 2:** Baseline characteristics of the depression‐free population and depression incidence developed during 2‐year follow‐up, *n* (%)

	Depression		*χ* ^2^/*t*	*p*
	Yes, *n* = 412	No, *n* = 3,872		
Age ($\bar{X}$ ± s)	55.4 ± 3.7	55.0 ± 3.6	2.05	.04
Female	1,593 (41.14)	240 (58.25)	44.54	.00
Education ($\bar{X}$ ± s)	12.1 ± 3.7	11.8 ± 3.7	1.71	.09
Married and living together with spouse	3,035 (78.38)	307 (74.51)	3.25	.07
BMI				
Underweight	48 (1.24)	7 (1.70)	1.32	.73
Normal	1,601 (41.37)	178 (43.20)		
Overweight	1,651 (42.66)	167 (40.53)		
Obese	570 (14.73)	60 (14.56)		
Any chronic diseases	2,194 (56.67)	265 (64.32)	8.93	.00
Any mobility limitation	908 (23.45)	132 (32.04)	14.92	.00
Less than good health	3,377 (87.22)	307 (74.51)	50.75	.00
Smokers	990 (25.58)	118 (28.64)	1.83	.18
Alcohol drinkers	514 (13.27)	41 (9.95)	3.65	.06
Vigorous or moderate physical activity	3,787 (97.80)	395 (95.87)	6.30	.04

Note

BMI: underweight (<18.5 kg/m^2^); normal (18.5–25 kg/m^2^); overweight (25–30 kg/m^2^); obese (≥30 kg/m^2^).

As shown in Table 3, high physical job demands (OR = 1.23) and high psychosocial job demands (OR = 1.36), but not low job control (OR = 1.18) were significantly related to a higher incidence of depression. A significant higher risk of depression was associated with high physical strain (OR = 1.39) and high psychosocial strain (OR = 1.55), but not with other categories of job strain after adjusting for all potential confounders. Similar results were found between job strain and depression incidence in the population without limiting the upper age to 63 years (aged 50–86).

**Table 3:** Odds ratio (OR) and 95% confidence interval (CI) of depression incidence in relation to job strain

	Depression‐free	Depression incidence	OR (95% CI)
	*n* = 3,872	*n* = 412	
Job demands (high vs. low)			
Physical demands	1,708 versus 2,164	205 versus 207	1.23 (1.00–1.52)
Psychosocial demands	1,881 versus 1,991	228 versus 184	1.36 (1.10–1.67)
Job control			
Low versus high	1,876 versus 1,996	222 versus 190	1.18 (0.96–1.46)
Physical strain			
Low	1,239 (32.0)	114 (27.7)	1.00
Passive	925 (23.9)	93 (22.6)	1.04 (0.78–1.39)
Active	757 (19.6)	76 (18.4)	1.08 (0.79–1.47)
High	951 (24.6)	129 (31.3)	1.39 (1.06–1.83)
Psychosocial strain			
Low	1,061 (27.4)	93 (22.6)	1.00
Passive	930 (24.0)	91 (22.1)	1.07 (0.79–1.46)
Active	935 (24.1)	97 (23.5)	1.24 (0.92–1.68)
High	946 (24.4)	131 (31.8)	1.55 (1.17–2.06)

Note

ORs were adjusted for age, gender, living arrangement, education, BMI, country, smoking, alcohol consumption, physical activity, chronic disease, mobility limitation, and health. The number of missing covariates was 237 of education, 118 of BMI, 116 of self‐reported health, 115 of age, gender, marital status, chronic disease, mobility, smoking, alcohol consumption, physical activity, and job strain, respectively.

The associations between baseline characteristics of the low physical strain population and high physical strain incidence developed during the 2‐year follow‐up were shown in Table 4. Incidence of high physical strain was higher in those individuals with lower education and poor self‐reported health than their respective counterparts. However, individuals with depression at baseline were not at higher risk of subsequent high physical strain.

**Table 4:** Baseline characteristics of population with lower physical strain and high physical strain incidence developed during 2‐year follow‐up, *n* (%)

Characteristics	Low physical strain, *n* = 909	Passive physical job, *n* = 865	Active physical job, *n* = 704	High physical strain, *n* = 781	*p*
Age ($\bar{X}$ ± s)	54.9 ± 3.5	54.6 ± 3.4	54.8 ± 3.4	54.9 ± 3.5	.09
Female	406 (44.7)	394 (45.5)	303 (43.0)	384 (49.2)	.10
Education ($\bar{X}$ ± s)	13.7 ± 3.4	12.9 ± 3.4	12.3 ± 3.5	11.8 ± 3.8	.00
Married and living together with spouse	695 (76.5)	663 (94.2)	514 (73.0)	603 (77.2)	.23
BMI					
Underweight	14 (1.5)	10 (1.2)	13 (1.8)	14 (1.8)	.79
Normal	406 (44.7)	382 (44.2)	288 (40.9)	343 (43.9)	
Overweight	364 (40.1)	349 (40.3)	309 (43.9)	311 (39.8)	
Obese	124 (13.7)	124 (14.3)	94 (13.4)	113 (14.5)	
Any chronic diseases	523 (57.5)	499 (57.7)	400 (56.8)	469 (60.1)	.60
Any mobility limitations	220 (24.2)	216 (25.0)	159 (22.6)	211 (27.0)	.25
Less than good	111 (12.2)	134 (15.5)	83 (11.8)	135 (17.3)	.00
Smoker	216 (23.8)	220 (25.4)	171 (24.3)	184 (23.6)	.81
Alcohol drinker	114 (12.6)	100 (11.6)	95 (13.5)	98 (12.5)	.72
Vigorous or moderate physical activity	879 (96.8)	843 (97.5)	691 (98.2)	758 (97.1)	.46
Depression	130 (14.3)	138 (16.0)	117 (16.6)	146 (18.7)	.11

Table 5 shows the relationships between baseline characteristics of the low psychosocial strain population and occurrence of high psychosocial strain during the 2‐year follow‐up. Incidence of high psychosocial strain was higher in younger, in male, in those with lower education, married and living together with spouse, and in those with at least one mobility limitation or less than good health. However, individuals with depression at baseline were not at higher risk of subsequent high psychosocial strain.

**Table 5:** Baseline characteristics of population with lower psychosocial strain and high psychosocial strain incidence developed during 2‐year follow‐up, *n* (%)

Characteristics	Low psychosocial strain, *n* = 801	Passive psychosocial job, *n* = 1,061	Active psychosocial job, *n* = 628	High psychosocial strain, *n* = 705	*p*
Age ($\bar{X}$ ± s)	55.1 ± 3.5	55.0 ± 3.6	54.5 ± 3.4	54.5 ± 3.3	.00
Female	384 (47.9)	506 (47.7)	254 (40.4)	311 (44.1)	.01
Education ($\bar{X}$ ± s)	12.8 ± 3.5	11.5 ± 3.8	13.2 ± 3.5	12.4 ± 3.7	.00
Married and living together with spouse	593 (74.0)	785 (74.0)	479 (76.3)	571 (81.0)	.00
BMI					
Underweight	12 (1.5)	16 (1.5)	9 (1.4)	11 (1.6)	.81
Normal	358 (44.8)	449 (42.3)	272 (43.3)	286 (40.6)	
Overweight	313 (39.1)	436 (41.1)	268 (42.7)	304 (43.1)	
Obese	117 (14.6)	160 (15.1)	79 (12.6)	104 (14.8)	
Any chronic diseases	461 (57.6)	619 (58.3)	350 (55.7)	416 (59.0)	.64
Any mobility limitations	172 (21.5)	292 (27.5)	142 (22.6)	196 (27.8)	.00
Less than good	101 (12.6)	183 (17.2)	75 (11.9)	112 (15.9)	.01
Smoker	176 (22.0)	269 (25.4)	165 (26.3)	194 (27.5)	.08
Alcohol drinker	105 (13.1)	130 (12.3)	88 (14.0)	86 (12.2)	.70
Vigorous or moderate	779 (97.4)	1,040 (98.0)	605 (96.3)	686 (97.3)	.29
Depression	113 (14.1)	166 (15.6)	104 (16.6)	119 (16.9)	.45

Due to the prevalence and incidence of depression as well as job strain were different across countries, logistic regression might not provide valid estimates, and multilevel analysis was employed to estimate job strain in predicting depression incidence while taking into account the country‐level differences (data not shown). Significant fixed intercept (Estimate = 2.31, *p* < .000) from the intercept‐only model (Model 0), suggested that the variance of the mean for each country were significantly different from the overall mean incidence of depression, and about 23.4% of the variance could be explained by country (ICC = 0.23). After adding job strain (level‐1 predictors), the proportion of variance could be explained by country (ICC) approximately increased to 0.24, suggesting although job strain varied by country, the associations between job strain and depression incidence were similar across countries. In the model that included both the country‐ and individual‐level factors (Model 1), high job strain (OR_phy‐strain_ = 1.65, OR_psy‐strain_ = 1.83) was still significantly related to a higher risk of depression incidence but not with other categories. These results were similar to those from logistic regression models, supporting the validity of using logistic regression in analyzing these data.

## 4 DISCUSSION

This study showed that using the same assessment instrument, the prevalence of depression varied across countries in working population. High physical demands, high psychosocial demands, high physical strain, and high psychosocial strain might independently increase depression incidence, while baseline depression was not related to subsequent high physical or psychosocial strain.

Few studies have been conducted on the prevalence of depression in working population using the same assessment instrument. In this cohort study of participants aged 50–63, we found the depression prevalence was 17.4%, which was comparable to the reported prevalence (ranging from 12.5% to 27.2%) in other studies (Copeland et al., 2004, 1999; Volkert, Schulz, Harter, Wlodarczyk, & Andreas, 2013). These variations could be due to cultural (Börsch‐Supan, Hank, & Jürges, 2005; Guerra et al., 2016) and linguistic differences in response to the wording in the questions included in the depression assessment instrument (Castro‐Costa et al., 2007), the differences in socioeconomic status and working conditions, as well as in rates of nonparticipants. It is understandable that people with depression are less willing to participate in surveys; in our study, participants had lower depression prevalence (13.96%) as compared with nonparticipants (38.87%).

In this study, we found that both physical demands and psychosocial job demands have similar association with the incidence of depression. These findings are in line with previous studies on the topic (Van Den Bogaard & Henkens, 2018; Wahrendorf et al., 2013). Although job demands‐control model has two dimensions, a number of studies have focused on job control only due to its higher sensitivity compared with job demands (Wang, Schmitz, Dewa, & Stansfeld, 2009; Wedegaertner et al., 2013). In contrast, some studies suggested a stronger association of depression with high psychosocial demands than with low job control (Bultmann, Kant, Schroer, & Kasl, 2002; Smith & Bielecky, 2012), arguing that job strain may be underestimated in previous studies. Our study examined both dimensions and found that participants with either high job physical demands and psychosocial demands or low job control were more likely to develop depression as compared to their respective counterparts. After adjusting for confounders, these associations became weaker with low job control but stronger with job physical demands and psychosocial demands. These findings were consistent with previous studies which reported that high psychosocial demands may be a stronger predictor for depression than low job control (Bultmann et al., 2002; Smith & Bielecky, 2012).

The current study found that both high physical strain and high psychosocial strain were significantly associated with depression incidence after adjusting for potential confounders. Similar results have been reported previously (Burns, Butterworth, & Anstey, 2016; Mezuk et al., 2011), and a study suggested reducing job strain may lower depression incidence (Wang et al., 2009). These results are also in line with a review which reported that 16/19 high‐quality studies supporting a causal relationships between work and health across time (De Lange, Taris, Kompier, Houtman, & Bongers, 2003).

The observed association between job strain and depression in the present study was further confirmed in multilevel analysis. Although only a few studies have taken into account country‐level influence in their analysis, these findings were supported by previous studies showing that depression was strongly associated with individual‐level factors rather than country‐level factors (Rai, Zitko, Jones, Lynch, & Araya, 2013; Stolz, Fux, Mayerl, Rasky, & Freidl, 2016).

Regarding gender difference, we found that high job strain was strongly associated with depression in male but not in female; however, previous studies reported that adverse working conditions are related to a similar increase in depression among men and women (Theorell et al., 2015, 2014). This could be explained by the fact that female had higher level job strain compared with male in one of the previous studies (Theorell et al., 2014), while female and male shared similar working environments in our study. The difference could also be interpreted by a wide age range of the participants in previous study (Theorell et al., 2015) while the participants in the present study are older workers. Indeed, because the gender differences in social roles, male might be more likely to act as the major bread‐winner role in a family (Tsai & Chang, 2016), performance at work might be more important to achieve self‐worth for male than female (Wang, Patten, Currie, Sareen, & Schmitz, 2012), even with similar levels of job strain, male would take it more seriously than female. Notably, due to gender differences in social roles, female used to have multitasks, and their threshold level of stress may be higher than male. Moreover, women more often take activities outside of work than men (Finkel, Andel, & Pedersen, 2016), so that they may have a better way to balance their emotions than male.

To our knowledge, this is one of the few studies that examined the reciprocal associations between job strain and depression incidence. A weaker relationship between work characteristics and mental health was found in previous study (De Lange et al., 2004), but not in our study, while previous study adjusted for age and gender only, we also adjusted for health‐related factors, because they were not only related to depression but also strongly associated with job strain (Mayerl, Stolz, Grossschadl, Rasky, & Freidl, 2017). Several explanations are possible. Since people with depression were more vulnerable to experience negative working conditions (Nigatu, Reijneveld, Penninx, Schoevers, & Bultmann, 2015), when the level of negative working condition was high, they would be more likely to leave the work, especially among those who have severe depression. In addition, people with depression were more likely to have poor health (Doom & Haeffel, 2013), leading to early leaving from their work (Wedegaertner et al., 2013), thus the participants in the analysis should have better health and less severe depression than nonparticipants. Health worker effect should also be pointed out, workers should be in better health compared with general working age population if they were employed; therefore, participants included in this study may have lower susceptibility to depression. Indeed, we found that among those who have low job strain at baseline, nonparticipants due to missing data on job strain were more likely to be older (60–63 years) and with higher levels of depression at the 2‐year follow‐up. These relatively healthy participants in this subpopulation could have been exposed to less risk factors compared with nonparticipants, which could underestimate the association between baseline depression and job strain incidence as these factors were strongly associated with depression (Fitch et al., 2017) and elevated job strain (De Lange et al., 2004; Shigemi, Mino, & Tsuda, 2000). On the other hand, individuals who recovered from depression and those who had effective treatment might have higher levels of tolerance to high job strain, so that they might not experience high strain even if the strain was high. More studies were warranted to confirm these results.

There are several strengths in the current study. First, this is one of the few studies that provides evidence on the reciprocal relationships between job strain and depression. Second, recall bias was minimized as the ascertainment of outcome occurred after the exposure assessment. Third, the relative large sample size permitted valid analysis. Fourth, the narrow age range in our study ensured better internal consistency. Moreover, additional multilevel analysis was performed to confirm the findings. Finally, data from a number of European countries increased the generalizability of the findings.

Nevertheless, some limitations should be considered. First, the interpretation of the results needs caution because unavailable information on duration of exposure to job strain and that longer duration of exposure to job strain might be more likely to affect depression than the shorter duration; thus, the associations can be either over or underestimated. Second, job strain and depression were estimated with self‐reported questionnaires, information bias might occur when any information regarding working conditions and depression was ascertained inaccurately. For example, a participant could either not be aware of exposure to job strain or over‐reporting job strain. Similarly, participants may erroneously report depression. Third, job strain was measured by the short battery, which may not accurately pick out individuals with high job strain. Final, we could not exclude the possibility that residual confounding might have biased the true association.

## 5 CONCLUSIONS

In conclusion, the prevalence of depression varies across countries. Baseline high demands but not low control may be associated with elevated risks of depression incidence, high job strain may further increase this risk. However, baseline depression may be not related to subsequent high job strain. To level off late‐life depression epidemic, protecting workers from high job strain can be an effective preventive strategy.

## CONFLICT OF INTEREST

None declared.

## AUTHOR CONTRIBUTIONS

Ya‐Mei Qiao, Ya‐Ke Lu, and Hui‐Xin Wang designed and conducted the study. Ya‐Mei Qiao and Ya‐Ke Lu analyzed the data, and Ya‐Mei Qiao drafted the manuscript. Hui‐Xin Wang, Zhen Yan, Wu Yao, and Jin‐Jing Pei involved in the interpretation of the results and critical revision of the manuscript.

## Data Availability

The data that support the findings of this study are openly available in SHARE wave 1 (Data set. https://doi.org/10.6103/SHARE.w1.611) and wave 2 (Data set. https://doi.org/10.6103/SHARE.w2.611), reference number (Börsch‐Supan, 2018a, 2018b; Börsch‐Supan et al., 2013, 2008; Börsch‐Supan & Jürges, 2005).
